# Loss of heterozygosity and microsatellite instability in hepatocellular carcinoma in Taiwan

**DOI:** 10.1038/sj.bjc.6690380

**Published:** 1999-05-01

**Authors:** J-C Sheu, Y-W Lin, H-C Chou, G-T Huang, H-S Lee, Y-H Lin, S-Y Huang, C-H Chen, J-T Wang, P-H Lee, J-T Lin, F-J Lu, D-S Chen

**Affiliations:** Departments of Internal Medicine, andSurgery, College of Medicine, National Taiwan University Hospital, No. 1, Chang-Te Street, Taipei, Taiwan; Department of Biochemistry, National Taiwan University, No. 7, Chung-Shan S. Road, Taipei, Taiwan

**Keywords:** loss of heterozygosity, microsatellite, hepatocellular carcinoma

## Abstract

Elucidation of the basic genetic changes of human hepatocellular carcinoma is important for the understanding and treatment of this cancer. We used microsatellite polymorphism markers to study 30 cases of hepatocellular carcinoma (34 tumours) on all human chromosomes. DNA from 34 pairs of hepatocellular carcinomas and corresponding non-tumour parts was prepared. Loss of heterozygosity (LOH) and microsatellite instability on 23 chromosomes were investigated by 231 sets of microsatellite markers. More than 20% LOH was shown for loci on 16q (47.1%), 13q (32.4%), 17p (32.4%), 5q (26.5%), 11p (23.5%) and 9p (20.6%). The commonly affected regions were mapped to 16q12.1, 16q12.2, 16q24, 13q12.1–32, 17p13, 5q32, 5q34, 5q3, 11p15, 11q23–24 and 9p21. Hepatitis B virus carriers had a significantly higher frequency of LOH on chromosomes 5q, 11p and 16q. Furthermore, larger tumour size tended to have higher frequency of LOH at D16S409 locus (16q12.1). Microsatellte instability was only found in 12 of 231 markers and the frequency is very low. These data suggest that the chromosomes 16q, 13q, 17p, 5q, 11p and 9p might participate in hepatocarcinogenesis. However, microsatellite instability might play little role in the development of this cancer in Taiwan. © 1999 Cancer Research Campaign


*British Journal of Cancer* (1999) **80**(3/4), 468–476
© 1999 Cancer Research Campaign
Article no. bjoc.1998.0380

### Loss of heterozygosity and microsatellite instability in hepatocellular carcinoma in Taiwan

**J-C Sheu[1], Y-W Lin[3], H-C Chou[1], G-T Huang[1], H-S Lee[1], Y-H Lin[1], S-Y Huang[1], C-H Chen[1], J-T Wang[1], P-H Lee[2], J-T Lin[1], F-J Lu[3] and D-S Chen[1]**

Departments of [1]Internal Medicine and [2]Surgery,College of Medicine, National Taiwan University Hospital, No. 1, Chang-Te Street, Taipei, Taiwan; [3]Department of Biochemistry, National Taiwan University, No. 7, Chung-Shan S. Road, Taipei, Taiwan

**Summary** Elucidation of the basic genetic changes of human hepatocellular carcinoma is important for the understanding and treatment of this cancer. We used microsatellite polymorphism markers to study 30 cases of hepatocellular carcinoma (34 tumours) on all human chromosomes. DNA from 34 pairs of hepatocellular carcinomas and corresponding non-tumour parts was prepared. Loss of heterozygosity (LOH) and microsatellite instability on 23 chromosomes were investigated by 231 sets of microsatellite markers. More than 20% LOH was shown for loci on 16q (47.1%), 13q (32.4%), 17p (32.4%), 5q (26.5%), 11p (23.5%) and 9p (20.6%). The commonly affected regions were mapped to 16q12.1, 16q12.2, 16q24, 13q12.1–32, 17p13, 5q32, 5q34, 5q3, 11p15, 11q23–24 and 9p21. Hepatitis B virus carriers had a significantly higher frequency of LOH on chromosomes 5q, 11p and 16q. Furthermore, larger tumour size tended to have higher frequency of LOH at D16S409 locus (16q12.1). Microsatellte instability was only found in 12 of 231 markers and the frequency is very low. These data suggest that the chromosomes 16q, 13q, 17p, 5q, 11p and 9p might participate in hepatocarcinogenesis. However, microsatellite instability might play little role in the development of this cancer in Taiwan.

**Keywords:** loss of heterozygosity; microsatellite; hepatocellular carcinoma

Hepatocellular carcinoma (HCC) is one of the most common malignancies in the world, particularly in Southeast Asia and in Sub-Saharan Africa (Linsell and Higginson, 1976; Beasley, 1982). In Taiwan, it ranked the first in cancer mortality. The disease is known to be closely associated with chronic hepatitis B or C viral infection (Beasley, 1982; Chen, 1987, Chen et al, 1990), or exposure to α-toxin $B_1$ and environmental factors (Sheu et al, 1992). Nevertheless, the molecular mechanism still needs to be clarified.

The genesis of human cancers is a multistep process reflecting cumulative genetic alterations that include activation of oncogenes or inactivation of tumour suppressor genes (Sheu et al, 1992; Weissenbach et al, 1992). Loss of heterozygosity (LOH) in polymorphic markers in a chromosomal fragment is known to be indicative of the presence of putative tumour suppressor genes (Yeh et al, 1994, 1996; Kuroki et al, 1995*a*).

The recently identified short tandem repeat, the microsatellite, which exhibits length polymorphism is widely and evenly distributed throughout the whole genome (Weissenbach et al, 1992). These microsatellite markers have been used for primary gene mapping and linkage analysis (Weissenbach et al, 1992). Polymerase chain reaction (PCR) amplification of these sequences allows rapid assessment of LOH and greatly simplifies the detection of LOH (Yeh et al, 1994, 1996; Kuroki et al, 1995*a*, 1995*b*). Studies based on somatic LOH as a means of identifying critical loci have already led to the discovery of several important tumour suppressor genes, including the retinoblastoma (Rb), BRCA1, BRCA2 and DPC4 genes (Kamb et al, 1994; Miki et al, 1994; Linardopoulos et al, 1995; Wooster et al, 1995; Hahn et al, 1996).

Identification of subtle genetic changes indicative of microsatellite instability can be obtained through the analysis of mobility change of tumour DNA in electrophoresis (Peinado et al, 1992; Ionov et al, 1993). Microsatellite instability has been reported in a variety of human cancers, including colorectal carcinomas, but not in adjacent normal tissues from the same individual (Peinado et al, 1992; Gonzalez-Zulueta et al, 1993; Han et al, 1993; Ionov et al, 1993; Peltomaki et al, 1993; Risinger et al, 1993; Thibodeau et al, 1993; Loeb, 1994; Merlo et al, 1994). In hereditary non-polyposis colorectal carcinoma (HNPCC), this microsatellite instability is apparently due to inherited and somatic mutations in mismatch repair genes (Loeb, 1994). Mutations of these microsatellite instability genes may be an important mechanism in tumorigenesis.

By using restriction fragment length polymorphism (RFLP) and microsatellite analysis, previous reports have demonstrated that allelic losses on chromosomes 1p, 4q, 5q, 8p, 8q, 11p, 13q, 16q and 17p are frequently found in HCC (Nose et al, 1993; Takahashi et al, 1993; Fujimoto et al, 1994; Fujiwara et al, 1994; Yeh et al, 1994, 1996; Kuroki et al, 1995*a*, 1995*b*; Yumoto et al, 1995; Nagai et al, 1997). These observations implicated that loss, or alterations, of a number of tumour suppressor genes in these loci might play an important role in the development of HCC. However, conflicting results regarding the frequency and site of chromosome loss have been shown in these studies. In the present study we used a total of 231 markers, representing 1–22 and X chromosomes to examine systematically the LOH frequency in 30 cases of HCC. At the same time, microsatellite instability was analysed. We hoped to determine the specific sites for the putative tumour suppressor genes in HCC. Meanwhile, the correlation with clinical parameters was also studied.

*Received 4 August 1998*
*Revised 12 November 1998*
*Accepted 17 November 1998*

*Correspondence to:* J-C Sheu

**Table 1** Clinical data of HCC patients

| Case no. | Sex | Age | HBsAg | α-fetoprotein (mg ml$^{-1}$) | Anti-HCV | Tumour diameter (cm) | Liver state[a] | LOH Percentage[b] | No. of instability |
|---|---|---|---|---|---|---|---|---|---|
| 1 | F | 55 | N | < 20 | P | 3.6 | Cir | 6.2 (11/178) | 0 |
| 2 | M | 41 | P | < 20 | N | 7 | Cir | 9.3 (14/151) | 0 |
| 3 | M | 59 | P | 139 | N | 2.6 | Cir | 1.3 (2/153) | 1 |
| 4 | F | 67 | N | < 20 | P | 1.8 | Cir | 0 (0/164) | 0 |
| 5 | M | 60 | P | 597 | N | 5 | Cir | 0.6 (1/168) | 0 |
| 6 | M | 64 | P | < 20 | ND | 5 | Cir | 7.5 (12/161) | 0 |
| 7 | M | 46 | P | 47 | N | 2.2 | Cir | 26.6 (42/158) | 2 |
| 8 | M | 67 | P | < 20 | N | 3.6 | CPH | 10 (15/150) | 1 |
| 9 | M | 39 | P | 25979 | ND | 8 | Cir | 6 (9/152) | 0 |
| 10 | M | 29 | P | 21598 | ND | 4.3 | Cir | 4.7 (7/150) | 0 |
| 11 | F | 43 | P | < 20 | ND | 4.5 | Non-Cir | 0 (0/164) | 1 |
| 12 | F | 68 | N | 646 | P | 4.5 | Cir | 0 (0/171) | 0 |
| 13 | M | 65 | P | < 20 | P | 2.2 | Cir | 3.2 (5/154) | 0 |
| 14 | F | 57 | N | 47192 | N | 7.5 | Cir | 9.3 (16/172) | 0 |
| 15 | M | 50 | P | < 20 | N | 2.5 | Cir | 0 (0/166) | 0 |
| 16 | M | 44 | P | 237 | ND | 5 | Cir | 7.1 (11/155) | 1 |
| 17 | F | 47 | P | < 20 | N | 5 | CPH | 16.8 (28/167) | 0 |
| 18 | F | 64 | N | 497 | P | 3 | Cir | 3.4 (5/147) | 0 |
| 19 | M | 31 | P | 190 | N | 4 | Cir | 10.4 (17/163) | 0 |
| 20 | M | 56 | P | < 20 | P | 17 | CPH | 2 (3/151) | 1 |
| 21 | F | 60 | N | < 20 | P | 4.8 | Cir | 0 (0/170) | 2 |
| 22 | M | 60 | N | 37 | P | 2.5 | Cir | 0 (0/152) | 0 |
| 23 | M | 60 | P | > 35000 | N | 20 | CPH | 0 (0/161) | 0 |
| 24 | M | 63 | N | 905 | P | 2 | Cir | 0 (0/182) | 0 |
| 25 | M | 63 | N | 23 | P | 3.2 | Cir | 0 (0/174) | 0 |
| 26 | F | 65 | P | 1995 | N | 7 | Cir | 11.9 (18/151) | 2 |
| 27 | M | 52 | P | 649 | N | 4 | Non-Cir | 0 (0/155) | 0 |
| 28 | M | 54 | P | < 20 | N | 13 | CPH | 0 (0/162) | 0 |
| 29 | M | 57 | P | < 20 | N | Ta = 3 | Cir | 32.3 (53/164) | 3 |
| | | | | < 20 | | Tb = 2 | | 0.6 (1/165) | 0 |
| | | | | 69.3 | | $T_1$ = 2 | | 9.7 (16/165) | 0 |
| | | | | 69.3 | | $T_2$ = 1.7 | | 6.1 (10/165) | 0 |
| 30 | M | 64 | N | < 20 | P | Ta = 2.8 | CPH | 1.3 (2/154) | 0 |
| | | | | | | Tb = 2.2 | | 0 (0/153) | 0 |

Ta, Tb: primary tumours; $T_1$, $T_2$: recurrent tumours; ND: not determined; F: female; M: male; N: negative; P: positive. [a]: Cir, cirrhosis; CPH, chronic persistent hepatitis. [b](no. of primers with LOH)(total no. of informative primers).

### MATERIALS AND METHODS

### Patients

Primary HCC tissues and their corresponding non-neoplastic liver tissues were obtained from 30 HCC patients receiving surgical resection in National Taiwan University. The diagnosis of HCC was confirmed by histology. Both tumour and non-tumour parts were frozen immediately after surgery and stored at −135°C until use. A total of 30 HCC patients were included in this study (Table 1). One patient (case 29) received two operations due to primary and recurrent HCCs, and there were two tumours in each operation. Another patient (case 30) had two small tumours. Therefore, a total of 34 tumours were studied. The tumour size was less than 3 cm in 14 tumours, between 3 and 5 cm in 13 tumours, and larger than 5 cm in the remaining seven tumours. Twenty-one patients were male and nine were female. The age ranged from 29 to 67 years. Twenty were positive and ten were negative for hepatitis B surface antigen (HBsAg). Hepatitis C virus antibody (anti-HCV) was positive in two of the 20 patients who were positive for HBsAg. However, in the ten patients negative for HBsAg, nine were positive and only one was negative for anti-HCV.

### DNA isolation

Genomic DNAs were extracted from the tumour and non-tumour liver tissues using methods described previously (Sheu et al, 1992).

### Microsatellite polymorphism analysis

All these samples, including tumour and non-tumour parts, were systematically studied by using the microsatellite markers (from chromosomes 1–22 and X chromosome). These markers were selected at a distance of every 10–20 cM according to Weissenbach (Weissenbach et al, 1992) (Table 2). The primers specific for these markers are commercially available (Research Genetics, Huntsville, AL, USA) (Weissenbach et al, 1992). A total of 231 microsatellite markers were used. Each marker was amplified by PCR in 25-μl volumes of a mixture containing 25 ng of genomic DNA template, 5 pmol of each primer, 75 μM each of deoxyguanosine triphosphate, deoxythymidine triphosphate and deoxycytidine triphosphate, 7.5 μM triphosphate deoxyadenosine triphosphate, 1.5 mM magnesium, 1X PCR buffer, 2.5 μCi of [$^{35}$S]dATP 5 U μl$^{-1}$, and 0.5 U *Taq*. PCR was performed in a thermocycler which can fit 96-microtiter plates (PTC-100–96V, MJ

© Cancer Research Campaign 1999

Research Inc., Watertown, MA, USA). Reactions were performed in 27 cycles under the following conditions: 30 s at 94°C for denaturation, 75 s at 55°C for primer annealing and 30 s at 72°C for primer extension. Finally, PCR product was further incubated in 72°C for another 6 min. The pipetting and dispensing of the PCR reagents was performed by a Robotic workstation (Biomek 2000, Beckman, Palo Alto, CA, USA). After completion of PCR, the PCR products were run on a 6% polyacrylamide gel electrophoresis (PAGE) gel followed by exposure of the gel to X-ray film for 2–3 days. The band pattern between tumour and non-tumour part was compared. The genetic changes, including LOH and microsatellite instability, were analysed. The assessment of LOH and microsatellite instability was done as described previously (Loeb, 1994; Yeh et al, 1994; Nagai et al, 1997)

### Statistical analysis

We correlate LOH frequency with several clinical aspects, including sex, age, tumour size, liver state, serum α-fetoprotein (AFP) and status of hepatitis B or C virus infection. The statistical analysis was performed by the computer program STATISTICA (StatSoft, Tulsa, OK, USA). The *P*-values were obtained by Fisher's exact tests and the *r*-values were obtained by linear regression analysis.

### RESULTS

### Study on LOH

We analysed 34 HCC samples and their corresponding non-tumour DNA for LOH by microsatellite markers from chromosomes 1–22 and X chromosome. A total of 231 markers were used in this study. The mean heterozygosity of the microsatellite markers was 70%. The representative results of microsatellite amplifications in HCCs are shown in Figure 1. In the DNA of non-tumour part that was heterozygous at a locus, two alleles were amplified at similar efficiency, and the resulting PCR product was separated on the gel as two clustering bands of equivalent intensity. In contrast, in tumour part with loss of one allele at this microsatellite locus, the gel showed disappearance of clustering bands indicating LOH.

#### *Frequency of LOH in each samples*

Among the 34 HCC samples analysed, the frequency of LOH (number of primers with LOH/number of informative primers) ranged from 0% to 32.2% (average 5.5%) (Table 1). Except for two HCC samples (case 7 and case 29 Ta) that had LOH frequency of 32.3% and 26.6%, most of the HCC samples had LOH frequency less than 10%. Of the 34 HCCs, 11 HCCs did not show any LOH with the 231 markers tested.

In the four tumours of case 29, who had two tumours in two operations, one tumour (Ta) had the highest LOH (53/164, 32.2%) and the other three tumours had LOH less than 10%. In the two tumours of case 30, who had two tumours, one tumour (Ta) had two LOH locus, but the other tumour (Tb) had no LOH. The sites of LOH in the four HCCs of the first case and the two HCCs of the second case were all different.

#### *Frequency of LOH in each chromosome*

When the data were analysed based on the frequency of LOH in each chromosome (allelic loss at one or more markers), the range of LOH was from 0% to 47.1% (average 15.2%). There were six chromosomes which had LOH more than 20%. The highest rate of LOH were on chromosome 16 (16/34, 47.1%), followed by 5 (12/34, 35.3%), 13 (11/34, 32.4%), 17 (11/34, 32.4%), 9 (8/34, 26.5%) and 11 (8/34, 23.5%) (Table 3).

#### *Frequency of LOH based on each microsatellite marker*

The percentage of LOH on all microsatellite markers for 34 HCC samples is shown in Table 2. The average frequency of LOH in each microsatellite locus ranged from 0% to 50% (average 5.4%). Most of the markers had LOH less than 20%, and only 12 markers had LOH more than 20%. Among the 231 markers, the highest rates of LOH were detected for specific loci on chromosome arm 9p21 (20.8%),

**Table 2** Microsatellite markers used in this study and results of LOH at each locus in 34 HCCs

| Chromosome | | | | | | | | | | | |
|---|---|---|---|---|---|---|---|---|---|---|---|
| 1 | 2 | 3 | 4 | 5 | 6 | 7 | 8 | 9 | 10 | 11 | 12 |
| D1S201 (0/23, 0%) | D2S158 (0/23, 0%) | D3S1307 (0/27, 0%) | D4S419 (1/21, 4.8%) | D5S406 (3/18, 16.7%) | D6S296 (0/25, 0%) | D7S511 (0/28, 0%) | D8S265 (0/6, 0%) | D9S168 (3/21, 14.3%) | D10S223 (0/23, 0%) | D11S932 (7/29, 24.1%) | D12S93 (1/26, 3.8%) |
| D1S211 (0/24, 0%) | D2S146 (0/34, 0%) | D3S1297 (1/29, 3.4%) | D4S396 (0/16, 0%) | D5S395 (0/14, 0%) | D6S289 (0/27, 0%) | D7S493 (0/26, 0%) | D8S282 (4/28, 14.3%) | D9S162 (1/20, 5%) | D10S197 (0/29, 0%) | D11S921 (2/27, 7.4%) | D12S89 (0/21, 0%) |
| D1S232 (0/25, 0%) | D2S123 (0/23, 0%) | D3S1304 (1/27, 3.7%) | D4S416 (1/23, 4.3%) | D5S426 (0/29, 0%) | D6S306 (0/27, 0%) | D7S485 (0/26, 0%) | D8S278 (2/25, 8%) | D9S169 (5/24, 20.8%) | D10S218 (2/23, 8.7%) | D11S915 (2/21, 9.5%) | D12S87 (0/13, 0%) |
| D1S200 (0/27, 0%) | D2S160 (1/27, 3.7%) | D3S1263 (1/27, 3.7%) | D4S410 (0/14, 0%) | D5S420 (0/34, 0%) | D6S272 (0/27, 0%) | D7S499 (1/27, 3.7%) | D8S260 (2/30, 6.7%) | D9S165 (3/25, 12%) | D10S206 (1/19, 5.3%) | D11S914 (2/21, 9.5%) | D12S96 (0/10, 0%) |
| D1S220 (0/32, 0%) | D2S114 (1/27, 3.7%) | D3S1266 (0/19, 0%) | D4S406 (2/18, 11.1%) | D5S427 (3/28, 10.7%) | D6S313 (1/18, 5.6%) | D7S502 (1/30, 3.3%) | D8S271 (0/25, 0%) | D9S197 (2/21, 9.5%) | D10S215 (0/17, 0%) | D11S905 (2/34, 5.9%) | D12S104 (0/27, 0%) |
| D1S230 (0/26, 0%) | D2S150 (1/24, 4.2%) | D3S1260 (0/20, 0%) | D4S427 (4/28, 14.3%) | D5S428 (1/23, 4.3%) | D6S284 (3/27, 11.1%) | D7S524 (0/20, 0%) | D8S269 (0/8, 0%) | D9S177 (1/21, 4.8%) | D10S192 (2/27, 7.4%) | D11S937 (2/28, 7.1%) | D12S92 (0/34, 0%) |
| D1S219 (0/26, 0%) | D2S142 (1/26, 3.8%) | D3S1313 (1/24, 4.2%) | D4S417 (2/15, 13.3%) | D5S421 (0/23, 0%) | D6S283 (4/28, 14.3%) | D7S501 (0/24, 0%) | D8S284 (0/30, 0%) | D9S179 (3/28, 10.7%) | D10S217 (2/29, 6.9%) | D11S938 (5/19, 26.3%) | D12S106 (0/1, 0%) |
| D1S236 (1/23, 4.3%) | D2S152 (1/26, 3.8%) | D3S1261 (1/19, 5.3%) | D4S621 (0/10, 0%) | D5S393 (1/31, 3.2%) | D6S266 (4/24, 16.7%) | D7S522 (1/26, 3.8%) | | | | D11S934 (4/34, 11.8%) | D12S81 (0/34, 0%) |
| D1S223 (0/15, 0%) | D2S172 (0/27, 0%) | D3S1290 (0/25, 0%) | D4S415 (4/29, 13.8%) | D5S413 (4/25, 16%) | D6S303 (2/18, 11.1%) | D7S487 (0/21, 0%) | | | | | D12S101 (0/16, 0%) |
| D1S194 (0/15, 0%) | D2S140 (1/30, 3.3%) | D3S1279 (0/26, 0%) | | D5S410 (1/23, 4.3%) | D6S304 (2/21, 9.5%) | D7S500 (1/30, 3.3%) | | | | | D12S78 (0/16, 0%) |
| D1S218 (0/25, 0%) | | D3S1275 (0/10, 0%) | | D5S412 (4/31, 12.9%) | D6S262 (2/26, 7.7%) | D7S483 (2/28, 7.1%) | | | | | |
| D1S242 (0/27, 0%) | | D3S1262 (0/28, 0%) | | D5S423 (1/22, 4.5%) | D6S308 (3/26, 11.5%) | | | | | | |
| D1S215 (0/24, 0%) | | D3S1288 (1/34, 2.9%) | | D5S400 (4/28, 14.3%) | D6S314 (3/17, 17.6%) | | | | | | |
| D1S240 (0/19, 0%) | | D3S1272 (1/21, 4.8%) | | D5S425 (2/23, 8.7%) | D6S311 (2/25, 8%) | | | | | | |
| D1S254 (0/12, 0%) | | | | D5S394 (0/22, 0%) | D6S305 (2/23, 8.7%) | | | | | | |
| D1S191 (0/25, 0%) | | | | | | | | | | | |
| D1S222 (0/25, 0%) | | | | | | | | | | | |
| D1S238 (0/22, 0%) | | | | | | | | | | | |
| D1S306 (1/26, 3.8%) | | | | | | | | | | | |
| D1S249 (0/27, 0%) | | | | | | | | | | | |
| D1S217 (0/24, 0%) | | | | | | | | | | | |
| D1S237 (0/19, 0%) | | | | | | | | | | | |
| D1S304 (0/16, 0%) | | | | | | | | | | | |
| D1S204 (0/17, 0%) | | | | | | | | | | | |

()%: indicates LOH cases/informative cases, LOH frequency. X[a]: informative cases are few on X chromosome.

*© Cancer Research Campaign 1999*

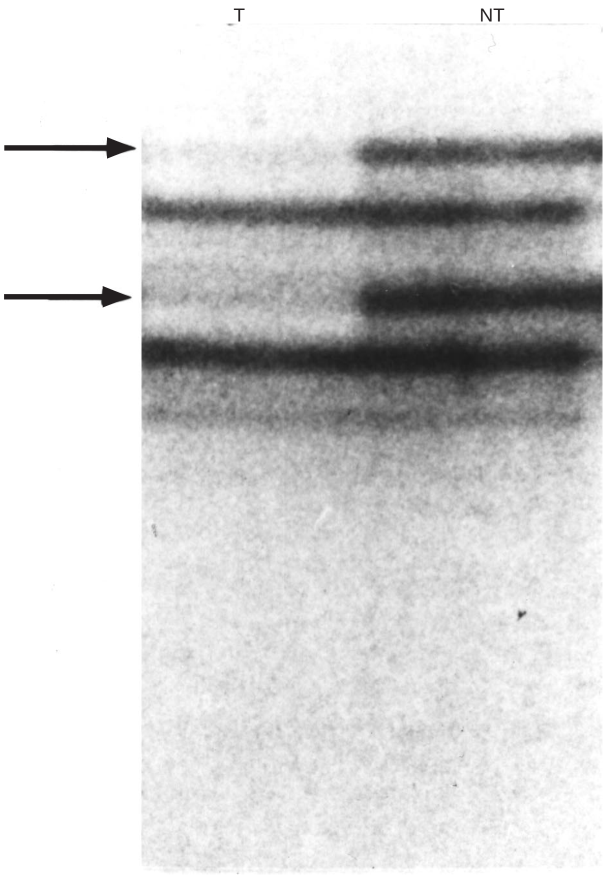

**Figure 1** A representative result of LOH in HCC. DNA from one pair of liver cancer (lane T) and non-tumour part (lane NT) was amplified by PCR with microsatellite primer D13S155. Tumour DNA showed a disappearance of one allele at this microsatellite loci

11p15 (26.3%), 11q23–24 (24.1%), 13q12.1–12.2 (25%), 13q14.3 (20.6%), 13q21–32 (31.3%), 16q12.1 (31.6), 16q12.2 (50%), 16q13 (33.3%), 16q24 (30.4–33.3%) and 17p13 (39.3%) (Table 4). The most frequent LOH was detected at locus D16S419 in 16q12.2 with a frequency of 50% of 14 informative HCCs in our study.

### *Deletion mapping of chromosomes with frequent LOH*

Deletion mapping by LOH analysis was performed on six chromosomes which had LOH frequency more than 20%. Deletion mapping of chromosome 16 is shown in Figure 2A. Most of the 16 tumours that had LOH span the q arm (16/34, 47.1%). By further mapping, the LOH regions were limited to three smaller regions. The first region was near marker D16S409, the second was near marker D16S419 and the third region is between markers D16S402 and 16S408. The locations of D16S409 and 16S419 were on chromosome 16q12.1 and 16q12.2, while the locations of D16S402 and D16S408 were on chromosome 16q24 and 16q13. From the LOH mapping of tumours 18, 19, 25 and 19, the third region was narrowed down to D16S422 (16q24). Therefore, the minimal LOH regions commonly affected in these patients were deduced to be 16q12.1, 16q12.2 and 16q24.

The deletion mapping of chromosome 13 is shown in Figure 2B. In most affected samples, the LOH regions cover 13q (11/34, 32.4%). Furthermore, we found a smaller region of LOH between D13S171 and D13S156. The locations of these markers were on chromosome 13q12.1 and 13q32.

For chromosome 17, the LOH region seemed to span the p arm (11/34, 32.4%) (Figure 2C). The minimal LOH region frequently affected in these 11 patients was deduced to be around marker D17S796. The localization of marker D17S796 was 17p13.

Similar analysis was performed on chromosomes 11, 9 and 5 (data not shown). Regions of LOH appear to cover both p arms of chromosome 11 (8/34, 23.5% for the p arms; 5/34, 15% for the q arms). In the p arm, the LOH region was near marker D11S932, while the q arm was between D11S938 and D11S934. The locations of D11S932 and D11S934 were on chromosome 11p15 and

| 13 | 14 | 15 | 16 | 17 | 18 | 19 | 20 | 21 | 22 | X[a] |
|---|---|---|---|---|---|---|---|---|---|---|
| D13S221 (1/15, 6.7%) | D14S72 (1/29, 3.4%) | D15S128 (3/30, 10%) | D16S418 (4/31, 12.9%) | D17S796 (11/28, 39.3%) | D18S59 (0/23, 0%) | D19S209 (3/23, 13%) | D20S113 (1/26, 3.8%) | D22S265 (0/28, 0%) | D22S280 (2/29, 6.9%) | DXS987 (0/7, 0%) |
| D13S171 (5/20, 25%) | D14S70 (0/29, 0%) | D15S165 (2/15, 13.3%) | D16S406 (2/30, 6.7%) | D17S786 (3/18, 16.7%) | D18S63 (0/29, 0%) | D19S221 (0/13, 0%) | D20S115 (0/18, 0%) | D21S269 (0/22, 0%) | D22S281 (2/25, 8%) | DXS989 (1/8, 12.5%) |
| D13S218 (2/24, 8.3%) | D14S75 (1/26, 3.8%) | D15S123 (1/30, 3.3%) | D16S414 (1/26, 3.8%) | D17S783 (1/17, 5.9%) | D18S62 (0/19, 0%) | D19S213 (0/25, 0%) | D20S104 (0/34, 0%) | D22S262 (1/29, 3.4%) | D22S278 (1/34, 2.9%) | DXS991 (0/7, 0%) |
| D13S155 (5/27, 18.5%) | D14S66 (1/24, 4.2%) | D15S119 (0/34, 0%) | D16S410 (2/18, 11.1%) | D17S805 (0/17, 0%) | D18S71 (0/27, 0%) | D19S220 (0/16, 0%) | D20S118 (0/34, 0%) | D21S261 (1/22, 4.5%) | D22S277 (2/34, 5.9%) | DXS1000 (0/4, 0%) |
| D13S153 (7/34, 20.6%) | D14S61 (1/24, 4.2%) | D15S125 (1/34, 2.9%) | D16S417 (1/19, 5.3%) | D17S791 (0/27, 0%) | D18S65 (1/28, 3.6%) | D19S223 (0/25, 0%) | D20S112 (0/34, 0%) | D21S270 (2/23, 8.7%) | D22S283 (1/33,3%) | DXS995 (1/9, 11.1%) |
| D13S176 (3/26, 11.5%) | D14S68 (2/23, 8.7%) | D15S127 (1/34, 2.9%) | D16S409 (6/19, 31.6%) | D17S788 (1/24, 4.2%) | D18S69 (1/32, 3.1%) | D19S217 (0/24, 0%) | D20S106 (0/25, 0%) | D21S267 (2/24, 8.3%) | D22S279 (1/34, 2.9%) | DXS990 (0/6, 0%) |
| D13S156 (5/16, 31.3%) | D14S81 (3/23, 13%) | | D16S416 (2/18, 11.1%) | D17S792 (0/24, 0%) | D18S60 (0/19, 0%) | D19S214 (0/26, 0%) | D20S120 (0/13, 0%) | D21S266 (2/33, 6.1%) | D22S276 (0/16, 0%) | DX984 (0/1, 0%) |
| D13S157 (2/20, 10%) | D14S78 (2/32, 6.3%) | | D16S419 (7/14, 50%) | D17S789 (1/14, 7.1%) | D18S68 (1/29, 3.4%) | D19S218 (0/28, 0%) | D20S100 (0/18, 0%) | | D22S282 (1/15, 6.7%) | |
| D13S159 (4/19, 21.1%) | | | D16S415 (4/25, 16%) | D17S784 (1/23, 4.3%) | D18S58 (3/28, 10.7%) | | D20S102 (0/34, 0%) | | D22S274 (0/33, 0%) | |
| D13S173 (1/30, 3.3%) | | | D16S408 (6/18, 33.3%) | | | | | | | |
| | | | D16S422 (8/24, 33.3) | | | | | | | |
| | | | D16S402 (7/23, 30.4%) | | | | | | | |

© Cancer Research Campaign 1999

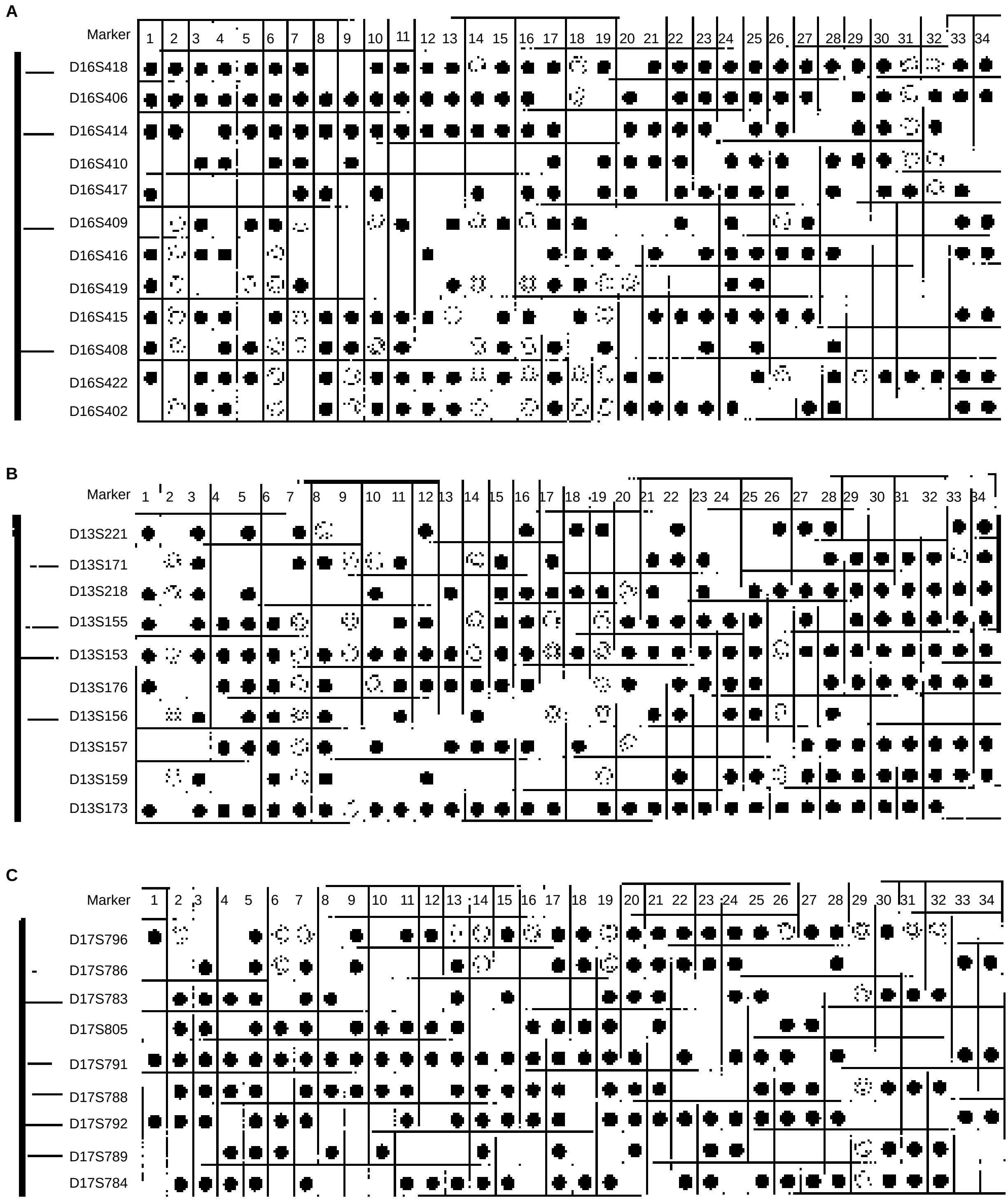

**Figure 2** Summary of the results of LOH analysis on chromosomes that have LOH frequency more than 15% in 34 HCCs. The locations of the microsatellite markers are shown. The patients are indicated by the number shown at the top of the Figure; case 29: tumours 29–32, case 30: tumours 33–34. Non-informative indicated no polymorphism between alleles at the markers. Therefore, no LOH information could be obtained. •, Informative with two alleles normally intact (●); LOH (○); non-informative (no allelic polymorphism) (□). (**A**) Chromosome 16, (**B**) chromosome 13, (**C**) chromosome 17

11q23–q24 respectively. For chromosome 9, the LOH regions spanned the whole 9p arm (7/34, 20.6%). One smaller LOH region was mapped to chromosome 9p21 (D9S169). For chromsome 5, we found that these LOH regions spanned the chromosome 5q arm (9/34, 26.5%). Three distinct regions, D5S413(5q32), D5S412(5q34) and D5S400(5q35.2), were mapped.

### *Correlation analysis based on HCC samples and LOH*

We correlated the LOH frequency of each HCC sample with the clinical status. There was no significant correlation with sex, age, status of HCV antibody, AFP or liver status. However, among the 34 HCC samples, tumours in those patients positive for HBsAg tended to have higher frequency of LOH (primers with LOH/

 © Cancer Research Campaign 1999

**Table 3** Summary results of LOH analysis in each chromosome in 34 HCC tumours

| Chromosome no. | 1 | 2 | 3 | 4 | 5 | 6 | 7 | 8 | 9 | 10 | 11 | 12 | 13 | 14 | 15 | 16 | 17 | 18 | 19 | 20 | 21 | 22 | X |
|---|---|---|---|---|---|---|---|---|---|---|---|---|---|---|---|---|---|---|---|---|---|---|---|
| No. of tumour with LOH (allelic loss at one more markers) | 1 | 4 | 2 | 6 | 12 | 4 | 4 | 6 | 9 | 2 | 8 | 1 | 11 | 5 | 3 | 16 | 11 | 3 | 3 | 1 | 2 | 3 | 2 |
| Frequency of LOH (%) | 2.9 | 11.8 | 5.9 | 17.6 | 35.3 | 11.8 | 11.8 | 17.6 | 26.5 | 5.9 | 23.5 | 2.9 | 32.4 | 14.7 | 8.8 | 47.1 | 32.4 | 8.8 | 8.8 | 2.9 | 5.9 | 8.8 | 5.9 |

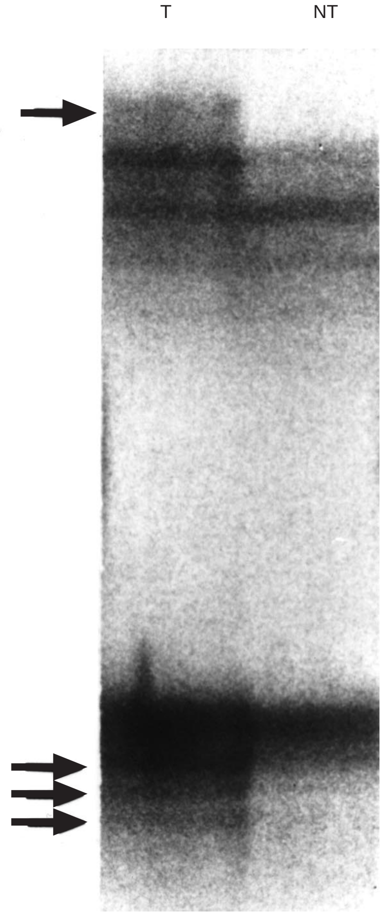

**Figure 3** A result of microsatellite instability in HCC. DNA from one pair of liver cancer (lane T) and non-tumour part (lane NT) was amplified by PCR with microsatellite primers D19S214. Arrows show increasing or decreasing number of repeats informative primers) (Table 1), but the difference was not statistically significant ($P > 0.05$)

### *Correlation analysis based on each chromosome and LOH*

After analysing several clinical aspects, including sex, age, AFP and status of HCV infection, no significant correlation between these parameters and LOH frequency for each chromosome was found. Interestingly, we found significant correlation between LOH on chromosome 5q, 11p, 16q and positivity of HBsAg.

For the 34 randomly selected HCCs, LOH on chromosome 5q was found in nine of 23 (39.1%) HCCs who were positive for HBsAg, but none in 11 (0%) HCCs who were HBsAg-negative. A significant difference between these two groups was observed ($P < 0.05$). At the same time, LOH on chromosome 11p was found more frequently in HBV-positive than in HBV-negative HCCs (8/23, 34.7% vs 0/11, 0%; $P < 0.05$). Similarly, a significant correlation between hepatitis B virus carrier and LOH on chromosome 16q was also found (14/23, 60.7% vs 2/11, 18%; $P < 0.05$).

**Table 4** Summary of microsatellite marker loci demonstrating significant frequency of LOH in HCC

| Chromosomal locations | Locus | LOH frequency | |
|---|---|---|---|
| | | LOH/informative cases | (%) |
| 9p21 | D9S169 | 5/24 | 20.8 |
| 11p15 | D11S932 | 7/29 | 26.3 |
| 11q23–24 | D11S938 | 5/19 | 24.1 |
| 13q12.1–q12.2 | D13S171 | 5/20 | 25.0 |
| 13q14.3 | D13S153 | 7/34 | 20.6 |
| 13q21–q32 | D13S156 | 5/16 | 31.3 |
| 16q12.1 | D16S409 | 6/19 | 31.6 |
| 16q12.2 | D16S419 | 7/14 | 50.0 |
| 16q13 | D16S408 | 6/18 | 33.3 |
| 16q24 | D16S422 | 8/24 | 33.3 |
| | D16S402 | 7/23 | 30.4 |
| 17p13 | D17S796 | 11/28 | 39.3 |

### *Correlation analysis based on each locus and LOH*

After analysing the clinical parameters with the LOH frequency that were greater than 20% in 13 microsatellite loci, we found the larger tumour size tended to have higher frequency of LOH at D16S409 ($r = 0.59136$, $P = 0.0097$). However, there was no significant correlation between the LOH frequency of the remaining 12 microsatellite loci and clinical parameters.

### Study on microsatellite instability

The mobility shift in tumour DNA compared to corresponding normal DNA samples representing microsatellite instability is shown in Figure 3. Except for nine HCC samples which had few numbers of instability (Table 1), most of the HCCs had no band shift on gel electrophoresis. Microsatellite instability was found in 12 of 231 markers: D1S219, D1S249, D4S406, D5S413, D7S502, D11S914, D13S155, D13S176, D19S217, D19S214, DX989 and DX990. Only one patient had instability on the marker for each locus respectively. Of the 12 markers which had microsatellite instability, ten showed an increasing number of repetitive sequences, the remaining two showed a decreasing number of repetitive sequences.

© Cancer Research Campaign 1999 

### DISCUSSION

Recently, microsatellite polymorphism studies have determined frequent LOH on chromosomes 1p, 1q, 2q, 4q, 5q, 6q, 8p, 8q, 9p, 9q, 10q, 11p, 13q, 14q, 16p, 16q, 17p, 17q and 22q in HCCs (Buetow et al, 1989; Tsuda et al, 1990; Konishi et al, 1993; Nose et al, 1993; Takahashi et al, 1993; Fujimoto et al, 1994; Fujiwara et al, 1994; Yeh et al, 1994; Kuroki et al, 1995*a* 1995*b*; Yumoto et al, 1995; Nagai et al, 1997). In agreement with their data, we demonstrated frequent LOH on chromosomes 5q, 9p, 11p, 13q, 16q and 17p in this study. In addition, we found a new location exhibiting a high rate of LOH on chromosome 11q23–24 (24.1%). Therefore, chromosome 11q23–24 might be a new location of tumour suppressor gene. Our study did not find frequent LOH on chromosome arms 1p, 1q, 2q, 4q, 8p, 8q, 9q, 14q, 16p, 17q and 22q as reported by previous investigators. The most likely possibility of the difference is that the tumour size in our study is smaller than in previous investigations. The larger tumours might have obtained more secondary genetic changes during tumour development. However, the use of different microsatellite markers might be another possibility of this discrepancy.

Yeh et al (1994) reported LOH (30%) on chromosome 1p in HCC in Taiwan and mapped a commonly affected LOH region to 1p35–36. However, all the tumours used in their study were larger than 5 cm. Comprehensive allelotyping analysis by Nagai et al (1997) found frequent LOH on chromosome 1p and 1q in small HCCs. In the present study, a total of 24 microsatellite markers were used for chromosome 1, but our data showed only 3.3% LOH on chromosome 1.

In this study, we showed that chromosome 16q had the highest LOH frequency among all the chromosomes studied and the common LOH regions were mapped to 16q12.1, 16q12.2 and 16q24, which are different from 16q22–23 reported by Yeh et al (1996) and 16q23–24 reported by Nagai et al (1997). E-cadherin gene, which was located at chromosome 16q22, has been reported to play important role in tumour progression in a variety of human cancers including HCC (Slagle et al, 1993) and might be a tumour suppressor gene in this region. However, our data suggest that alterations occur in two different loci on 16q; this has not been reported in previous investigations. Because chromosome 16q contains many other important genes, such as phosphorylase kinase β (PHKβ, 16q12.1), retinoblastoma-related human gene (RB2, 16q12.2), collagenase (MMP2, 16q13), cytochrome c oxidase subunit IV (COX4, 16q24.1), adenine phosphoribosyl transferase (APRT, 16q24.2), *N*-acetylgalactosamine-6-sulphatase (GALNS, 16q24.2) and cellular adhesion regulatory molecule (CARM, 16q24.3), the deletion of this region may interfere with cell growth or function (Buetow et al, 1989; Tsuda et al, 1990; Slagle et al, 1993; Yeung et al, 1993; Kamatani, 1996). Therefore, it is likely that the two new loci detected in our study may contain important tumour suppressor genes.

As in our study, previous investigators (Kuroki et al, 1995*b*) found a high rate of LOH on 13q and mapped the common regions to 13q12.1–q32, which harbours the Rb and BRCA2 tumour suppressor genes (Hada et al, 1996). The role of these two genes in HCC needs to be established. Meanwhile, frequent LOH on chromosome 17p13 was also detected. The tumour suppressor gene, p53, located around chromosome 17p13 has been reported to be mutated frequently in a variety of human cancers including HCC (Hsu et al, 1994; Yumoto et al, 1995). In this region, p53 might be the target gene and play an important role in the development of HCC. As for chromosome 11, we mapped the minimal LOH region to 11p15 and 11q23–q24. Wang and Royler (1988) have mapped the fine LOH region to 11p13 in HCC, which was different from our data. The significance of these two new loci on chromosome 11 needs further study. Recently, the p16/MTS1 and P15/MTS2 genes, located at chromosome 9p21–22, were found to be important tumour suppressor genes in human cancers (Kamb et al, 1994; Linardopoulos et al, 1995). In this study, we found the LOH frequency was 14.7% at marker D9S169 on chromosome 9p21 in HCC. In addition, we found that the frequency of p15 gene alteration is about 14% in HCC (Lin et al, 1998). However, the frequency of p16 gene alteration is very rare (3%) in HCC in our studies (Lin et al, 1998) and in a previous report (Kita et al, 1996). These results suggested that p15 might be the tumour suppressor gene on 9p21 in HCC. However, the possibility of the presence of other tumour suppressor genes cannot be excluded.

Early studies have demonstrated high frequency of LOH on chromosome 4q in HCC (Yeh et al, 1996; Fujimoto et al, 1994). Because chromosome 4q contains genes encoding albumin, alcohol dehydrogenase (ADH3), fibrinogen and UDP-glucuronyltransferase, which are expressed predominantly in the liver, the LOH of this region may interfere the liver function (Buetow et al, 1989). Therefore, chromosomal alterations in this region seem to be an unique trait in HCC (Nagai et al, 1997). By microsatellite analysis, LOH on 4q has been mapped to 4q11–q12, 4q12–23, 4q22–24 and 4q35 (Buetow et al, 1989; Yeh et al, 1996; Nagai et al, 1997). However, our data showed lower LOH (14.3%) than previous data, in wider locus of 4q21–28 in HCC. Recently, Nagai et al (1997) reported that 42% of their HCCs had LOH on 8p23 (8S277) and argued that a tumour suppressor gene for HCC lies in this region. A candidate tumour suppressor gene, the PRLTS gene located on 8p21–22, has been found to be mutated in sporadic colorectal carcinoma and HCCs (Fujiwara et al, 1995). On the other hand, we also found LOH on 8p21.3 (D8S282) in only 14.3% of our HCCs. These differences might be due to different race, different tumour stages and different markers. However, further elucidation of the discrepancy is necessary.

As for chromosome 6, a candidate tumour suppressor gene, the M6P/IGF2R gene, which was mapped to 6q26–27, was found to have LOH in 70% of HCC (De Souza et al, 1995*a*). Recently, De Souza et al (1995*b*) showed that the M6P/IGF2R gene was deleted and mutated in HCCs with LOH. In our study, we only found LOH in 17.6% of our HCCs on 6q16.3–27 (D6S314), which includes the locus 6q26–27. The cause of this discrepancy needs further investigation. However, it is probable that the LOH in our series might be underestimated; recently, Yamada et al (1997) have shown that hepatocytes adjacent to HCCs that appear normal might already have LOH. The only way to elucidate the difference is to use blood or some other non-liver normal tissue for determining whether a person is informative at loci on 6q. This experiment is now ongoing in our lab.

HBV infection has been regarded as an important factor in the development of HCC (Beasley, 1982; Chen, 1987). However, the molecular mechanism is unclear. In this study we found the LOH frequency in HBsAg-positive HCC patients was significantly higher on chromosome 5q, 11p and 16q than those who are negative for HBsAg. These data imply that HBV infection might cause genetic changes within chromosomal regions where this is important for the development of some HCCs. Using RFLP, Wang and Rogler (1988) have demonstrated that allelic loss in 11p and 13q occurs in a higher percentage of HCCs arising from HBV carriers. Recently, Becker

 © Cancer Research Campaign 1999

and Zhou (1996) have shown frequent loss of chromosome 8p in HBV-positive HCC from China. The discrepancy needs further study. By linear regression, the LOH frequency of D16S409 (16q12.1) has a positive relation with tumour size. Previous investigation has reported that the high frequency of LOH on chromosome 16q arm is usually accompanied with advanced stage of HCC (Tsuda et al, 1990). Our data suggest that a tumour suppressor gene near D16S409 (16q12.1) might be involved in the progression of HCC. In addition to HBsAg and tumour size, we found no correlation between LOH frequency and clinical parameters, including sex, AFP, age, liver state and anti-HCV.

To study the role of microsatellite instability in liver carcinogenesis, we screened 34 HCCs at 231 microsatellite loci on 22 autosomal chromosomes and X chromosome. Although microsatellite instability is significant in HNPCC and other cancers (Peinado et al, 1992; Gonzalez-Zulueta et al, 1993; Han et al, 1993; Ionov et al, 1993; Peltomaki et al, 1993; Risinger et al, 1993; Thibodeau et al, 1993; Loeb, 1994; Merlo et al, 1994), in our study, microsatellite instability was only found in 12 of 231 markers. More recently, Kazachokov et al (1998) and Macdonald et al (1998) have shown that microsatellite instability occurs in approximately 35% of HCCs. The discrepancy in microsatellite instability between our HCCs and their HCCs might reflect the difference in the tumour size (smaller in our series) and the fact that the number of markers we used was more extensive. However, other factors such as different geographic area and different criteria for microsatellite instability might contribute to the difference.

In this study, one patient (case 29) had multiple and recurrent tumours, and the other patient (case 30) had multiple tumours. After analysing the six multiple small HCCs, we found all the tumours had different sites of LOH. Although the case number is limited, these data further support our previous observations that multiple small HCCs usually arise from different clonality (Sheu et al, 1993).

In summary, we have used microsatellite polymorphism analysis to screen LOH frequency of every chromosome in HCC, and the results provide a basal information for further positional cloning of putative tumour suppressor genes. Further fine mapping of LOH on chromosome arms that have a higher frequency of LOH, and cloning of the candidate genes in these areas, is ongoing in our lab.

### ACKNOWLEDGEMENTS

This study was supported by grants from Department of Health and National Science Council, Executive Yuan and Liver Disease Prevention and Treatment Research Foundation, Taiwan, ROC.

### REFERENCES

Beasley RP (1982) Hepatitis B virus as the etiologic agent in hepatocellular carcinoma-epidemiologic considerations. *Hepatology* **2**: 21–26

Becker SA and Zhou YZ (1996) Frequent loss of chromosome 8p in hepatitis B virus-positive hepatocellular carcinomas from China. *Cancer Res* **56**: 5092–5097

Buetow KH, Murray JC, Israel JL, London WT, Smith M, Kew M, Blanquet V, Brechot C, Redeker A and Govindarajah S (1989) Loss of heterozygosity suggests tumour suppressor gene responsible for primary hepatocellular carcinoma. *Proc Nat Acad Sci USA* **86**: 8852–8856

Chen DS (1987) Hepatitis B virus infection, its sequelae, and prevention in Taiwan. In: *Neoplasms of the Liver*, Okuda K and Ishak KG (eds), pp. 71–80. Springer-Verlag: Tokyo

Chen DS, Kuo GC, Sung JL, Lai MY, Sheu JC, Chen PJ, Yang PM, Hsu HM, Chang MH, Chen CJ, Hahn LC, Choo QL, Wang TH and Houghton M (1990) Hepatitis C virus infection in an area hyperendemic for hepatitis B and chronic liver disease: the Taiwan experience. *J Infect Dis* **162**: 817–822

De Souza AT, Hankins GR, Washington MK, Fine RL, Orton TC and Jirtle RL (1995*a*) Frequent loss of hetrozygosity on 6q at the mannose 6-phosphate/insulin-like growth factor II receptor locus in human hepatocellular tumors. *Oncogene* **10**: 1725–1729

De Souza AT, Hankins GR, Washington MK, Orton TC and Jirtle RL (1995*b*) M6p/IGF2R gene is mutated in human hepatocellular carcinoma. *Nat Gen* **11**: 447–449

Fujimoto Y, Hampton LL, Wirth PJ, Wang NJ, Xie JP and Thorgeirsson SS (1994) Alterations of tumor suppressor genes and allelic losses in human hepatocellular carcinomas in China. *Cancer Res* **54**: 281–285

Fujiwara Y, Ohata H, Emi M, Okui K, Koyama K, Tsuchiya E, Nakajima T, Monden M, Mori T and Kurimasa A (1994) A 3-Mb physical map of the chromosome region 8p21.3–p22, including a 600-kb region commonly deleted in human hepatocellular carcinoma, colorectal cancer, and non-small cell lung cancer. *Genes Chromosomes Cancer* **10**: 7–14

Fujiwara Y, Ohata H, Kuroki T, Koyama K, Tsuchiya E, Monden M and Nakamura Y (1995) Isolation of a candidate gene on chromosome 8p21.3–22 that is homologous to an extracellular domain of the PDGF receptor beta gene. *Oncogene* **10**: 891–895

Gonzalez-Zulueta M, Ruppert, JM, Tokino K, Tsai YC, Spruck CH3d, Miyao N, Nichols PW, Hermann GG, Horn T, Steven K (1993) Microsatellite instability in bladder cancer. *Cancer Res* **53**: 5620–5623

Hada H, Koide N, Morita T, Shiraha H, Shinji T, Nakamura M, Ujike K, Takayama N, Oka T, Hanafusa T, Yumoto Y, Hamazaki K and Tsuji T (1996) Promoter-independent loss of mRNA and protein of the Rb gene in a human hepatocellular carcinoma. *Hepatogastroenterology* **43**: 1185–1189

Hahn SA, Schutte M, Hoque AT, Moskaluk CA, da Costa LT, Rozenblum E, Fischer A, Yeo CJ, Hruban RH and Kern SE (1996) DPC4, a candidate tumor suppressor gene at human chromosome 18q21.1. *Science* **271**: 350–353

Han HJ, Yangisawa A, Kato Y, Park JG and Nakamura Y (1993) Genetic instability in pancreatic cancer and poorly differentiated type of gastric cancer. *Cancer Res* **53**: 5087–5089

Hsu HC, Peng SY, Lai PL, Sheu JC, Chen DS, Lin LI, Slagle BL and Butel JS (1994) Allelotype and loss of heterozygosity of p53 in primary and recurrent hepatocellular carcinomas. A study of 150 patients. *Cancer* **73**: 42–47

Ionov Y, Peinado MA, Malkhosyan S, Shibata D and Perucho M (1993) Ubiquitous somatic mutations in simple repeated sequences reveal a new mechanism for colonic carcinogenesis. *Nature* **363**: 558–561

Kamatani N (1996) Adenine phosphoribosyltransferase (APRT). *Nippon Rinsho Jpn J Clin Med* **54**: 3213–3219

Kamb A, Gruis NA, Weaver-Feldhaus J, Liu Q, Harshman K, Tavtigian SV, Stockert E Day RS III, Johnson BE and Skolnick MH (1994) A cell cycle regulatory potentially involved in genesis of many tumor types. *Science* **264**: 436–440

Kazachokov Y, Yoffe B, Khaoustov VI, Solomon H, Klintmalm GBG and Tabor E (1998) Microsatellite instability in human hepatocellular carcinoma: relationship to p53 abnormalities. *Liver* **18**: 156–161

Kita R, Nishida N, Fukuda Y, Azechi H, Matsuoka Y, Komeda T, Sando T, Nakao K and Ishizaki K (1996) Infrequent alterations of the $p16^{INK4A}$ gene in liver cancer. *Int J Cancer* **67**: 176–180

Konishi M, Kikuchi-Yanoshita R, Tanaka K, Sato C, Tsuruta K, Maeda Y, Koike M, Tanaka S, Nakamura Y, Hattori N (1993) Genetic changes and histopathological grades in human hepatocellular carcinomas. *Jpn J Cancer Res* **84**: 893–899

Kuroki T, Fujiwara Y, Tsuchiya E, Nakamori S, Imaoka S, Kanematsu T, and Nakamura Y (1995a) Accumulation of genetic changes during development and progression of hepatocellular carcinoma: loss of heterozygosity of chromosome arm 1p occurs at an early stage of hepatocarcinogenesis. *Genes Chromosomes Cancer* **13**: 163–167

Kuroki T, Fujiwara Y, Nakamori S, Imaoka S, Kanematsu T and Nakamura Y (1995b) Evidence for the presence of two tumour-suppressor genes for hepatocellular carcinoma on chromosome 13q. *Br J Cancer* **72**: 383–385

Lin YW, Chen CH, Huang GT, Lee PH, Wang JT, Chen DS, Lu FJ and Sheu JC (1998) Infrequent mutations and no methylation of *CDKN2A*(*MTS1*/*p16*) and *CDKN2B*(*MTS2*/*p15*) in hepatocellular carcinoma in Taiwan. *Eur J Cancer* **34**: 1789–1795

Linardopoulos S, Street AJ, Quelle DE, Parry D, Peters G, Sherr CJ and Balmain A (1995) Deletion and altered regulation of $p16^{INK4}$ and $p15^{INK4b}$ in undifferentiated mouse skin tumors. *Cancer Res* **55**: 5168–5172

© Cancer Research Campaign 1999

Linsell DA and Higginson J (1976) The geographic pathology of liver cell cancer. In *Liver Cell Cancer*, Cameron HM, Linsell DA and Warwick GP (eds), pp. 1–16. Elsevier: New York

Loeb LA (1994) Microsatellite instability: marker of a mutator phenotype in cancer. *Cancer Res* **54**: 5059–5063

Macdonald GA, Greenson JK, Saito K, Cherian SP, Appelman HD and Boland CR (1998) Microsatellite instability and loss of heterozygosity at DNA mismatch repair gene loci occurs during hepatic carcinogenesis. *Hepatology* **28**: 90–97

Merlo A, Mabry M, Gabrielson E, Vollmer R, Baylin SB and Sidransky D (1994) Frequent microsatellite instability in primary small cell lung cancer. *Cancer Res* **54**: 2098–2101

Miki Y, Swensen J, Shattuck-Eidens D, Futreal PA, Harshman K, Tavtigian S, Liu Q, Cochran C, Bennett LM, Ding W, Bell R, Rosenthal J, Hussey C, Tran T, McClure M, Frye C, Hattier T, Phelps R, Haugen-Strano A, Katcher H, Yakumo K, Gholami Z, Shaffer D, Stone S, Bayer S, Wray C, Bogden R, Dayananth P, Ward J, Tonin P, Narod S, Bristow PK, Norris FH, Helvering L, Morrison P, Rosteck P, Lai M, Barrett JC, Lewis C, Neuhausen S, Cannon-Albright L, Goldgar D, Wiseman R, Kamb A and Skolnick MH (1994) A strong candidate for the breast and ovarian cancer susceptibility gene BRCA1. *Science* **266**: 66–71

Nose H, Imazeki F, Ohto M and Omata M (1993) p53 gene mutations and 17p allelic deletions in hepatocellular carcinoma from Japan. *Cancer* **72**: 355–360

Nagai H, Pineau P, Tiollais P, Buendia AM and Dejean A (1997) Comprehensive allelotyping of human hepatocellular carcinoma. *Oncogene* **14**: 2927–2933

Peinado MA, Malkhosyan S, Velazquez A and Perucho M (1992) Isolation and characterization of allelic losses and gains in colorectal tumors by arbitrarily primed polymerase chain reaction. *Proc Natl Acad Sci USA* **89**: 10065–10069

Peltomaki P, Lothe RA, Aaltonen LA, Pylkkanen L, Nystrom-Lahti M, Seruca R, David L, Holm R, Ryberg D, Haugen A (1993) Microsatellite instability is associated with tumors that characterize the hereditary non-polyposis colorectal carcinoma syndrome. *Cancer Res* **53**: 5853–5855

Risinger JI, Berchuck A, Kohler MF, Watson P, Lynch HT and Boyd J (1993) Genetic instability of microsatellites in endometrial carcinoma. *Cancer Res* **53**: 5100–5103

Sheu JC, Huang GT, Shih LN, Lee WC, Chou HC, Wang JT, Wang JT, Lee HS and Chen DS (1992) Hepatitis C and B viruses in hepatitis B surface antigen negative hepatocellular carcinoma. *Gastroenterology* **103**: 1322–1327

Sheu JC, Huang GT, Chou HC, Lee PH, Wang JT and Chen DS (1993) Multiple hepatocellular carcinoma at the early stage have different clonality. *Gastroenterology* **105**: 1471–1476

Slagle BL, Zhou YZ, Birchmeier W and Scorsone KA (1993) Deletion of E-cadherin gene in hepatitis B virus-positive Chinese hepatocellular carcinomas. *Hepatology* **18**: 757–762

Takahashi K, Kudo J, Ishibashi H and Niho Y (1993) Loss of heterozygosity in hepatocellular carcinoma. *Nippon Rinsho Jpn J Clin Med* **51**: 370–374

Thibodeau SN, Bren G and Schaid D (1993) Microsatellite instability in cancer of the proximal colon. *Science* **260**: 816–819

Tsuda H, Zhang WD, Shimosato Y, Yokota J, Terada M, Sugimura T, Miyamura T and Hirohashi S (1990) Allele loss on chromosome 16 associated with progression of human hepatocellular carcinoma. *Proc Natl Acad Sci USA* **87**: 6791–6794

Wang HP and Rogler CE (1988) Deletions in human chromosome arms 11p and 13q in primary hepatocellular carcinomas. *Cytogen Cell Gen* **48**: 72–78

Weissenbach J, Gyapay G, Dib C, Vignal A, Morissette J, Millasseau P, Vaysseix G and Lathrop M (1992) A second-generation linkage map of the human genome. *Nature* **359**: 794–801

Wooster R, Bignell G, Lancaster JA, Swift S, Seal S, Mangion J, Collins N, Gregory S, Gumbs C and Micklem G (1995) Identification of the breast cancer susceptibility gene BRCA2. *Nature* **378**: 789–792

Yamada T, De Souza AT, Finkelstein S and Jirtle RL (1997) Loss of the gene encoding mannose 6-phosphate/insulin-like growth factor II receptor is an early event in liver carcinogenesis. *Proc Natl Acad Sci USA* **94**: 10351–10355

Yeh SH, Chen PJ, Chen HL, Lai MY, Wang CC and Chen DS (1994) Frequent genetic alterations at the distal region of chromosome 1p in human hepatocellular carcinomas. *Cancer Res* **54**: 4188–4192

Yeh SH, Chen PJ, Lai MY and Chen DS (1996) Allelic loss on chromosomes 4q and 16q in hepatocellular carcinoma: association with elevated alpha-fetoprotein production. Gastroenterology **110**: 184–192

Yeung RS, Bell DW, Testa JR, Mayol X, Baldi A, Grana X, Klinga-Levan K, Knudson AG and Giordano A (1993) The retinoblastoma-related gene, RB2, maps to human chromosome 16q12 and rat chromosome 19. *Onogene* **8**: 3465–3468

Yumoto Y, Hanafusa T, Hada H, Morita T, Ooguchi S, Shinji N, Mitani T, Hamaya K, Koide N and Tsuji T (1995) Loss of heterozygosity and analysis of mutation of p53 in hepatocellular carcinoma. *J Gastroenterol Hepatol* **10**: 179–185

*© Cancer Research Campaign 1999*
